# Effect of a community based social marketing strategy on the uptake of clean delivery kits in peri-urban communities of Karachi, Pakistan

**DOI:** 10.1186/s12884-022-04705-z

**Published:** 2022-05-28

**Authors:** Asra Usmani, Shazia Sultana, Imran Nisar, Shehla Zaidi, Imtiaz Jehan, Anita Zaidi

**Affiliations:** 1 Department of Pediatrics, The Aga Khan University, Karachi, Pakistan; 2 Department of Community Health Sciences, The Aga Khan University, Karachi, Pakistan

**Keywords:** Maternal mortality, Home delivery, Social marketing, Clean delivery kit

## Abstract

**Background:**

Pakistan has one of the highest neonatal and maternal mortality rates in the world. Use of clean delivery kits (CDK) at time of delivery improves maternal and newborn outcome. We test effectiveness of a social marketing strategy to increase uptake of CDKs in a low socioeconomic peri-urban community in Pakistan.

**Methods:**

This was a sequential mixed method study. The quantitative component consisted of two arms. In the prospective intervention arm trained community health workers (CHWs) visited pregnant women twice to prepare them for birth and encourage use of CDKs. Availability of these kits was ensured at accessible stores in these communities. The retrospective control arm consisted of women delivering in same area during the past 3 months identified from pregnancy register. Information was collected on sociodemographic, pregnancy characteristics and use of CDKs at time of delivery in both arms. We compared proportion of women using CDKs during home deliveries in the intervention and control arm. We performed logistic regression analysis to identify factors associated with use of CDKs in intervention arm. We carried out separate focused group discussions (FGDs) with women who used CDKs, with women who did not use CDKs and birth attendants.

**Results:**

Total of 568 pregnant women were enrolled in prospective intervention arm and 603 in retrospective control arm. The proportion of women using CDKs during home deliveries in retrospective control arm was 9.4% compared to 23.8% in prospective control arm (*p* =  < 0.001). In final multivariable model, increasing age of pregnant woman and husband having some education was positively associated with CDK use (aOR 1.1;95% CI 1.1–1.2 and aOR 2.2;95% CI 1.3–3.6 respectively). During FGDs, many women were of the thought that kits should be free or included in the amount charged by birth attendants. Assembly of components of kit into one package was appreciated by birth attendants.

**Conclusion:**

Social marketing strategy targeting pregnant women and their family members resulted in an increase in the uptake of CDKs in our study. Birth attendants were generally satisfied with the assembly of the kit. Many women cited unawareness and cost to be a major impediment in use of CDKs.

## Introduction

Around 295 000 women die every year globally during and following the period of pregnancy and childbirth. Most of these deaths (94%) occur in low-resource settings with South Asia accounting for nearly one-fifth of all deaths [1–3]. At the same time 2.4 million children die annually in the first month of life [4]. Pakistan has one of the highest maternal mortality ratio (MMR) and neonatal mortality rate (NMR) in the region, with a MMR of 140 per 100,000 live births and a NMR of 41 per 1000 live births [5, 6]. Although Pakistan is a signatory to the United Nation’s Sustainable Development Goals (SDGs) which sets to reduce MMR to less than 70 per 100 000 births and neonatal mortality to at least 12 per 1,000 live births by 2030, it is unlikely to meet both targets [7]. Both of these are considered few of the most sensitive indicators of a country’s health system and are closely monitored globally to mark progress in the domain of health and development [8, 9]. Besides being part of SDGs, NMR and MMR are important indicators included in Countdown 2005, 2015, 2017 and 2030 for women’s, children’s, and adolescent’s health [10, 11].

Most of the maternal deaths occur during labor or in the first 24 h postpartum while neonatal deaths are concentrated in the first week of life [12–14]. This period thus provides a window of opportunity to systematically address maternal and neonatal mortality and morbidity. Several interventions in the peripartum period have been shown to be effective in reducing maternal and neonatal mortality [15]. Some of these include increasing ANC coverage, birth preparedness, delivery in a health facility in the presence of a skilled birth attendant and adequate care in the post-natal period. Moreover, these work in a highly synergistic fashion when implemented together in packages [16]. Several modelling estimates have shown that when achieved sufficient coverage, the package of interventions can reduce the mortality in low-income and middle-income countries (LMICs) by 30–65% in both mothers and children [17, 18].

One component of birth preparedness necessitates that women deliver in the presence of a skilled birth attendant (SBA) in a clean, hygienic environment that reduces the chances of infection in both mother and child [19, 20]. The use of clean delivery kits (CDK) by SBAs has been widely encouraged in this regard [21, 22]. The CDKs are simple, disposable kits, containing soap for cleaning hands and perineum, a plastic sheet to provide a clean delivery surface for birth, a new razor blade for cord cutting and clean cord ties packaged in a box with illustrated instruction for use [23]. Despite being shown to be highly effective, the uptake of CDKs has been slow reaching to a mere 31.4% as per the Pakistan Demographic and Health survey (PDHS) 2006–7 [24]. This is in spite of existing recommendations by World Health Organization (WHO) on their routine use by birth attendants [25]. Studies on strategies to increase their uptake are lacking. Social marketing strategies have been successfully used to increase uptake of other health interventions like modern contraceptives, immunization and breastfeeding [26, 27]. Social marketing is defined as “the use of marketing to design and implement programs to promote socially beneficial behavior change” [28].

Here we evaluate, the effect of a community based social marketing strategy, comprising of two home visits by community health workers (CHWs) to pregnant women in the last trimester, on the uptake of safe delivery kits for use in home deliveries, in peri-urban communities of Karachi, Pakistan. In addition, we explore factors constraining or facilitating the uptake of these kits.

## Methodology

### Study setting

**Fig. 1:**
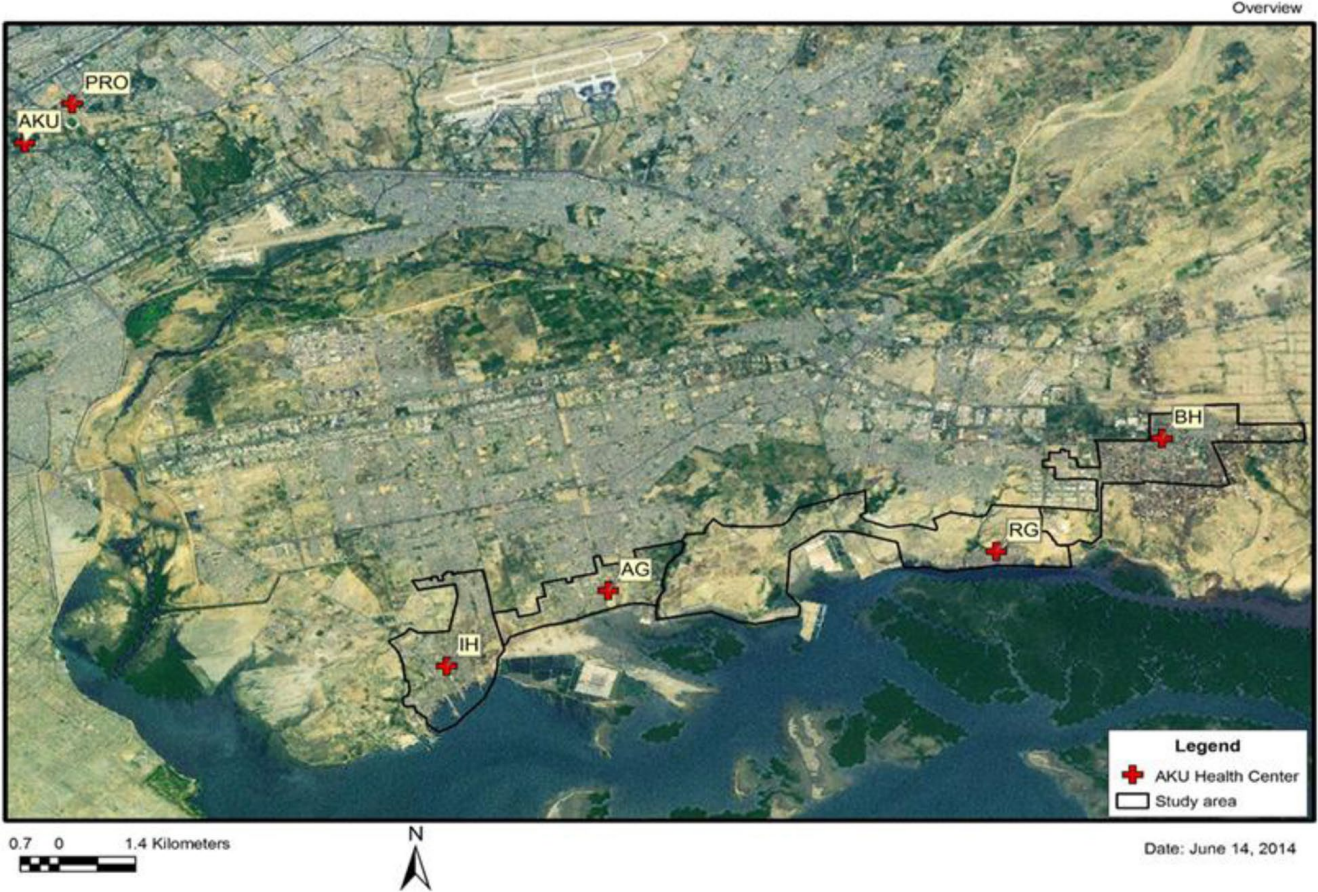
Map of study site. We have permission to reuse this image [29]

This study was conducted from October 2009 to March 2010 in four peri-urban communities, Rehri Goth, Ibrahim Hyderi, Bilal colony and Bhains colony in Karachi, Pakistan [29] (Fig. 1). These are low socioeconomic fishing communities and semi urban settlements.. The total population at the time of study was 212,294. Major occupations in this area are fishing, subsistence farming and small businesses. Most of the pregnant women deliver at home-in the presence of a traditional birth attendant (TBA), a close relative, or a skilled birth attendant (midwife or a nurse).

### Study design

This was an explanatory sequential mixed method study with a quantitative component followed by a qualitative component. The quantitative study consisted of a prospective intervention arm in which a cohort of pregnant women received the social marketing intervention. All women who were at least 24 weeks pregnant and were planning to deliver in the catchment area were eligible to be enrolled in the intervention arm. These were identified from an ongoing pregnancy surveillance at the study sites. Pregnant women in the intervention arm received two social marketing visits during the third trimester of the pregnancy. The target audience were the pregnant woman, her husband, and any other decision maker of the family. The 4 Ps of social marketing (Price, Place, Promotion, Product) were kept in view [30]. During the home visits, locally resident trained CHWs having at least 10 years of education, fluent in local languages and well-accepted by the community, provided information and education on the use and importance of CDKs. The time and date of sessions were scheduled as per the participants’ convenience. Pre-assembled CDKs were made available at convenient and accessible spots in the community (the Place). The contents of the kit, it's availability at the nearest *kiryana* (local store), its method of use, and cost effectiveness in preventing both maternal and newborn complications were discussed (the Product and the Price). Visual illustrations were used for explaining many of the concepts (the Promotion). Women were followed till the end of the pregnancy to record outcome of the pregnancy. Primary outcome was use of CDK’s in women who delivered at home. The control arm consisted of a retrospective cohort of pregnant women, selected from a pregnancy registry maintained at the sites and who had delivered in previous three months. They did not receive the social marketing intervention. They were visited during the study period to collect information on their pregnancy and use of CDKs.

The qualitative component consisted of separate focused group discussions (FGDs) with three distinct groups: women who bought CDKs and used in their home deliveries, women who did not buy CDKs and the birth attendants.

### Data collection

Data was collected during home visits by trained CHWs. In the intervention arm data was collected over a baseline enrolment visit, two additional visits during the pregnancy and one after the delivery. In the retrospective control arm, data was collected through one enrollment visit and covered all the information about the preceding pregnancy and its outcome. In both intervention and control arm, questions were asked on sociodemographic characteristics, past medical and obstetric history of the mother, use of CDKs and information on events around the peri-partum period. Specific questions were asked about the place of delivery, person assisting the delivery and key decision makers in the family for seeking care during and after pregnancy. If CDKs were used, women were asked about the items that were most used. If CDKs were not used, mothers were inquired about the reasons for not using them.

A total of 6 FGD’s were planned. Two were conducted with mothers in the intervention arm who used the CDK, two with mothers in the intervention arm who did not use the CDK and two FGDs were conducted with the birth attendants. Each FGD had around 5–7 participants and was conducted by the principal investigator herself along with a sociologist. Audio recording were made, and detailed notes were taken.

### Data management

Data was collected on paper forms and subsequently dual entered by two independent data entry operators using a customized program developed on Epi info version 6.04 (CDC, USA). Consistency and range checks were built in. A dual entry validation check was performed, and disagreements were resolved by going back to the paper forms.

### Sample size

Based on unpublished data from the ongoing surveillance at the study sites, we took baseline uptake of CDKs to be 10% in deliveries taking place at home (65% of the total deliveries). We calculated the sample size for a 15% absolute increase in proportion of women using the kits enrolled in the intervention arm and delivering at home, at 90% power and alpha of 0.05. Inflating for 15% lost to follow up in the intervention arm, we needed a minimum of 600 outcomes in the intervention and control arm.

### Plan of analysis

We describe distribution of the characteristics of the study participants by the study arm in which they were enrolled. Our primary outcome was proportion of women who used CDKs in the intervention and control arm. To identify predictors of uptake of CDK in the intervention arm, we performed multiple logistic regression. All variables which had a p-value of less than 0.10 in the bivariate analysis were included in the initial model. Backward elimination method was used to derive a parsimonious model with all variables having a p-value ≤ 0.05. Odds ratios with 95% confidence intervals are reported. Hosmer and Lemeshow test was used to assess the final fit of the model. All analysis was done using SPSS version 16.

For the qualitative arm of the study, coding was done for the responses of the participants of the FGDs by the principal investigator. Grounded theory was followed for coding plan. Codes helped summarize, synthesize, and sort observations or statements from the data which provided foundation for data analysis. Themes were identified after reviewing data at least thrice. Reporting was done of selected quotations and analysis of repeated themes.

While the qualitative and quantitative data were analyzed separately, the results were converged to compare and contrast findings during the final interpretation.

## Results

There were 568 women enrolled in the prospective intervention arm and 603 in the retrospective control arm. Women in the intervention arm were slightly younger (mean age 26.8 years versus 27.5 years; *p*-value = 0.022), had lower parity (2.8 versus 3.7; *p*-value < 0.001). A greater proportion of husbands in the control arm were health workers (7.6 vs 0.4%; *p*-value < 0.001). Roughly the same proportion of women in both arms delivered at home (63.6% versus 65.4%; *p*-value = 0.457) (Table 1). Among women who delivered at home, a significantly higher proportion of women used the CDK in the intervention arm as compared to the control arm (23.8% versus 9.4%; *p*-value < 0.001) (Table 2). The most common reason cited for not using CDK’s in the control arm was that women never heard about it (89.9%). In the final multivariable model, increasing age of the pregnant woman was positively associated with CDK use (aOR 1.1; 95% CI 1.1 to 1.2 for every one-year increase in age of the pregnant woman). Husband having some education was also positively associated with CDK use by the pregnant woman (aOR 2.2; 95% CI 1.3 to 3.6). (Table 3).

FGDs yielded a wealth of information. Some of the most important areas and their associated sub-themes, are summarized below and in Table 4.

**Table 1:** Socio demographic characteristics of study subjects

	Retrospective control arm *n* = 603	Prospective intervention arm *n* = 568	*P*-value
**Age of woman in years (mean ± SD)**	27.5 ± 5.4	26.8 ± 5.1	0.022
**Parity (mean ± SD)**	*n* = 603 3.7 ± 2.5	*n* = 566 2.8. ± 2	< 0.001
**Woman’s education status**	*n* = 603	*n* = 568	0.454
No education	478 (79.2%)	426 (75.0%)	
Up till Primary	93 (13.8%)	95 (16.7%)	
More than primary	42 (6.9%)	47 (8.3%)	
**Husband’s education status **	*n* = 601	*n* = 568	
No education	404 (67.2%)	380 (67.2%)	
Up till Primary	108 (18.0%)	111 (19.5%)	0.639
More than primary	91 (15.1%)	77 (13.5%)	
Husband’s occupation	*n* = 601	*n* = 568	< 0.001
Fisherman	281 (46.6%)	288 (50.7%)	
Shopkeeper	98 (16.3%)	62 (10.9%)	
Vendor	61 (10.1%)	43 (7.6%)	
Hospital worker	46 (7.6%)	2 (0.4%)	
Unemployed	17 (2.8%)	12 (2.1%)

**Table 2:** Home deliveries and use of clean delivery kits in the reference and intervention arm

Place of Birth	Retrospective control arm *n* = 603 n (%)	Prospective intervention arm *n* = 568 n (%)	*P*-value
	***n* = 603**	***n* = 567**	0.457
Home	382 (63.6%)	371 (65.4%)	
Health facility	221 (36.7%)	196 (34.6%)	
**Clean delivery kits used at home delivery**	*n* = 381	*n* = 370	< 0.001
Yes	36 (9.4%)	88 (23.8%)	
**Kits bought for use**	*n* = 36	***n* = 88**	** < 0.001**
Bought	25 (69.4%)	87 (98.9%)	
**Reason for not using the CDK**	***n* = 345**		
Never heard of it	310 (89.9%)	N/A	
Did not know where available	20 (5.8%)	N/A	
Birth attendant did not suggest	7 (2.0%)	N/A	
Lack of money	5 (1.4%)	-	
Did not like	3 (0.9%)	-	
**Had you bought if known**	*n* = 343		
Yes	286 (83.3%)	-

**Table 3:** Logistic regression model for uptake of clean delivery kits in home deliveries

Variables	Univariate analysis OR (95% CI)	Multivariate analysis aOR (95% CI)
Age of women	1.1 (1.1, 1.2)	1.1 (1.1,1.2)
Parity	1.2 (1.0,1.4)	-
Respondent’s education		
No education	Ref	Ref
Some education	2.16 (1.3, 3.6)	-
Husband’s education		
No education	Ref	Ref
Some education	2.2 (1.3,3.6)	2.2 (1.3,3.6)
Husband’s occupation		
Other	Ref	-
Factory worker	3.7 (2.2,6.3)	-
Fisherman	0.48 (0.2,0.6)	-
Previous use of clean delivery kit	2.1 (0.8,5.5)	-

**Table 4:** Focus group themes and sub themes

Research question	Sub themes
Reasons underlying non-use of the clean delivery kits	Lack of trust in new products
	Need to pay separate charge of kit
	Reluctance to adapt new practice due to no previous difficulty without CDK
Reasons underlying usage	Effective way of spreading information and creating awareness regarding clean delivery kits
	Ease of storage as clean delivery kits had all useful items in a single packet
	Clean delivery kit was available at accessible stores and at affordable price
Response of birth attendants	No objection from birth attendants towards the clean delivery kits
	Inconsistent promotion of kits has been a problem for the birth attendants previously

### FGDs with mothers who did not use CDKs

Most of the women cited lack of trust in a new product as a main reason for not using CDKs. They were cynical about the strategies applied to promote the kits. One woman said, *“We are illiterate; we have been fooled by many marketing campaigns, now we avoid new products.” (ID.3 FGD 1).* Another main reason included additional cost associated with purchasing a CDK and a perceived low risk of any complication with not using a CDK. They also mentioned that the money could instead be used for arranging a one-time meal for the family. *“We don’t have a regular source of income; we could have bought the kit if we had money; we could have even gone for hospital delivery but at that time we literally had no money.” (ID 1 FGD 2).* Some women cited their previous uneventful experience in the absence of using a CDK as reasoning for not using it in their most recent pregnancy. *“I gave birth to six kids at home without any such kit why should I use the kit this seventh time?” (ID 4 FGD2).* When asked about suggestions to improve the uptake of CDK, their spontaneous answer was to reduce the cost of the kit. Some of the participants were not satisfied with the strategy used as in their view it created a mistrust with the birth attendants. One participant added, *“Women should be concerned about pregnancy and delivery as far as diet and rest is concerned; technical things should not be their headache.” (ID 4 FGD 6).*

### FGDs with mothers who used CDKs

Women who used CDKs in the intervention arm were appreciative of the way intervention was delivered. i.e., CHWs creating awareness among their household including key decision makers like mother-in-law and husband. They thought that these approaches were more useful than passive campaigns like posters or advertisements on electronic media. Many had preexisting concerns about cleanliness during the birthing process and the associated tools used. *“We are always trying to keep our home clean, eat clean food and stay healthy but cleanliness during birth is generally neglected and ignored.” (ID 1 FGD3).* Women were attracted by the packaging of all the necessary items in one kit which subsidized the cost and was easily available from nearby stores. The packaging in a single kit also made the storage and retrieval of the items at time of need easier in-home settings where single items could be easily misplaced. Although the cost was still substantial for the community, many were willing to bear the cost given the explained benefits of CDKs. *“Fifty rupees are nothing if the benefits of the kit are kept in mind” (ID 4 FGD 4).*

### FGDs with birth attendants

In all cases, birth attendants reported having no objections to the use of CDKs, had previously used the kits and expressed satisfaction with the content of the kit. They found the sheet in the kit to be extremely useful in providing a clean surface for delivery especially in home surroundings which were very unclean. *“If kits are easily available then either woman can buy and keep at homes or we can take along at the time of delivery, it solves a lot of problems” (ID 3 FGD 6).* Birth attendants raised concerns about inconsistent distribution strategies deployed for CDKs in the past when they were sometimes provided free of cost to either the woman or birth attendant at a subsidized rate. This in turn affected their dealing with clients. One of the birth attendants said, *“I lost one of my clients when I tried to sell the kit. She had seen free distribution in her last delivery” (ID 1 FGD 5).*

## Discussion

Our study shows that a community based social marketing strategy was effective in increasing the uptake of CDKs during home deliveries in a low socioeconomic peri-urban setting. The mixed method design enabled us to explore factors that facilitated or hindered the use of CDK by the women or their birth attendants. We saw a 250% increase in the uptake of CDKs with social marketing. This complements existing literature on use of social marketing to increase the uptake of other health and behavioral interventions. [26–28]. A higher proportion of husbands in the retrospective control arm were health care workers. Since this would increase the uptake of CDKs in the control arm, the actual effectiveness of social marketing might be higher than what was observed.

Previous studies have highlighted the unique nature of pregnancy to be particularly effective at facilitating uptake of various health interventions. Pregnant women are more likely to seek out information regarding aspects of pregnancy and child birth than patients facing an illness [31–33]. Most of them also want to be involved in decision-making [34, 35]. A prospective case control study conducted in Iran showed significant reduction in unnecessary elective caesarian births amongst first time mothers who were delivered a social marketing campaign [36]. Similarly, social marketing was effectively used to increase breastfeeding in North East England [37].

In our study, the cost of CDKs was reported as a hindrance by the highest proportion of non-users and was also the most common concern raised in FGDs, often deemed as an extra expense or lesser priority than other materials. Earlier in Bangladesh a simple, affordable birth kit developed, and field tested to sell to pregnant women in rural areas showed that cost was an impediment. This led the investigators to plan to redesign the kit in order to reduce the cost [38].

After adjusting for other variables, age of the woman and education of the husband were found to be significantly associated with use of CDKs in home deliveries. Many a times it was the husband, in laws or other elders of the family who were the principal decision makers. These findings are consistent with those reported by a recent study that examined the factors affecting uptake of CDKs in Nepal. It revealed that inadequate knowledge of CDKs and lack of awareness about their potential benefits appear to be the main barriers to utilization in resource poor settings. Furthermore, household decision makers such as husbands and mothers-in-law are in a strong position to be able to promote or prevent CDK use, while women of reproductive age have less power within the household [39].

One strength of our study is that we show effectiveness of a social marketing strategy to increase uptake of CDKs in a setting with high maternal and neonatal mortality and homebirths. To the best of our knowledge, this is the first study evaluating the effect of social marketing on the uptake of CDKs in an LMIC setting. Although several studies have evaluated CHW involvement to improve maternal and infant birth outcome using an array of interventions, this is the first study to use social marketing by CHWs to increase uptake of CDKs in Pakistan. The study also had some limitations, including the lack of a concurrent comparison group given the nature of intervention and delivery. However, we overcame this by taking CDK uptake rates during preintervention period as a comparison group.

## Conclusion

We conclude that social marketing strategy delivered by CHWs targeting pregnant women and their family members can play an important role in improving use of CDKs in home deliveries. However, many women expect that the cost of the kit to be included in charges paid to birth attendants. Price, distribution points, target audience and stake holder’s involvement should be carefully considered when devising a social marketing strategy to ensure consistent and increased use of these kits in home deliveries. A combined approach targeting both pregnant women and their families as well as birth attendants may improve CDK use and be sustainable in the long run.

## Data Availability

All data supporting the findings of this article are available on request by contacting corresponding author Muhammad Imran Nisar at imran.nisar@aku.edu.

## Abbreviations

MMR: Maternal mortality ratio
NMR: Neonatal mortality rate
SDG: Sustainable Development Goals
CHW: Community health worker
CDK: Clean delivery kit
FGD: Focused group discussion
SBA: Skilled birth attendant
LMIC: Low-income and middle-income countries
